# Shared goals, communication and mutual respect in multicultural staff teams: A relational coordination perspective

**DOI:** 10.1002/nop2.704

**Published:** 2020-12-01

**Authors:** Laila Tingvold, Mai C. Munkejord

**Affiliations:** ^1^ Centre for Care Research East Norwegian University of Science and Technology Gjøvik Norway; ^2^ NORCE ‐ Norwegian Research Centre Bergen Norway

**Keywords:** communication, culture, multicultural teams, nurses, nursing, nursing homes

## Abstract

**Aim:**

Task interdependence among staff in nursing homes is high, and staff teams are typically composed of employees from different cultural backgrounds with varying levels of skills and qualifications. By applying relational coordination theory as an analytic lens, we investigated how communication and cooperation challenges are experienced and dealt with.

**Design:**

This is a qualitative study.

**Method:**

In‐dept interviews with 31 members of staff in two nursing homes and thematic analysis of the material.

**Results:**

The study identified challenges in direct communication and collaboration difficulties related to understanding how to use professional discretion in daily care practices in both nursing homes. The management in one of the two nursing homes arranged frequent meetings to share knowledge about the residents, their care status and introduced initiatives to build competence among all members of staff, including substitute and temporary staff. This contributed to ensuring shared goals and mutual respect at the workplace.

## 1 INTRODUCTION

Long‐term care (LTC) services in many Western countries are constrained by major challenges. First, care services must respond to the demographic development by providing welfare services to an increasing number of older people (Hussein, 2011; Musick & Wilson, 2008). Second, municipal health and care services across the industrialized world have undergone major organizational changes over the past years. In Norway, where this study was conducted, reforms have transferred responsibility for different user groups from the national and the regional levels to the municipal level and municipalities are now responsible for providing LTC services (Gautun & Syse, 2017; Otnes, 2015; Romøren, 2018). Moreover, after surgery, patients are discharged early from the hospital and transferred to their home municipality where they are supposed to receive necessary services**—**either in their own home, or if necessary and available, in a local nursing home. Thus, nursing home residents typically live with multimorbidity and polypharmacy and in addition, they often experience cognitive decline and/or mental disorders (Helvik et al., 2010; Selbæk et al., 2008). These trends lead to increased complexity of tasks in LTC services (Gautun & Syse, 2013; Tingvold & Magnussen, 2018). Moreover, staff members meet increasingly higher expectations with regard to skills in communication as well as in elderly care, dementia care and in geriatrics (Bing‐Jonsson et al., 2016; Tyrholm et al., 2016).

Despite this, approximately one of three person‐years in the municipal healthcare services in Norway is currently carried out by “unskilled” workers (Tjerbo et al., 2012). Included in this category are staff members with no formal health and care qualifications and who are new to the field, as well as persons who have worked as assistants in LTC for many years but without having obtained certification as a skilled care worker (as yet). Many of these “unskilled” workers, in particular among those recruited in recent years, are immigrants. By immigrants we mean persons born in a foreign country of two foreign parents (Statistics Norway, 2018). In fact, due to predicted staff shortages in the near future, there has been considerable political interest in Norway for the last 15–20 years in recruiting migrant workers in LTC (Helse‐ og omsorgsdepartementet, 2006, 2013). Also, in Norway today, in general one fifth of all nursing home staff are immigrants (SSB, 2018). Moreover, among newly recruited staff members, as many as 40% of the registered nurses (RNs) and more than 50% of the auxiliary nurses and unskilled assistants, are immigrants (Munkejord, 2017).

Internationally, several studies have, not surprisingly, identified communication difficulties among immigrant members of staff (Egede‐Nissen et al., 2017; Ho & Chiang, 2015; Magnusdottir, 2005; Xu et al., 2008). Some studies indicate that staff with an immigrant background may have a divergent understanding of tasks and prioritization compared with employees of majority background (Bourgeault et al., 2010; Durant & Shepherd, 2009; Yi & Jezewski, 2000). Concerns have been shared as to whether immigrant workers are becoming deskilled and undervalued as they experience problems in getting their qualifications and education recognized abroad (Adhikari & Melia, 2015; Kurniati et al., 2017; Salami & Nelson, 2014). On the other hand, studies have detected that good management or “diversity management” (which refers to organizational actions that aim to promote greater inclusion of employees from different backgrounds into an organization's structure through specific policies and programmes) can help to curb negative outcomes and counteract ethnically based workplace discrimination and deskilling (Janssens & Zanoni, 2014; Munkejord, 2019; Munkejord & Tingvold, 2019; Prasad et al., 2006).

## 2 RELATIONAL COORDINATION

To enter the LTC sector as an RN or as an auxiliary nurse in Norway, a high command of the Norwegian language is required, but to start working as an assistant, no formal national requirements exist. Thus, especially among immigrants working as assistants in nursing homes, the additional challenge of being a language learner adds to the complex working situation briefly described above. Against this background, managers in nursing homes are faced with the demanding task of coordinating staff teams composed of workers with different backgrounds and different competencies. In fact, coordination of staff members is known to be essential to the accomplishment of good services and patient outcomes (Gittell et al., 2000; Manser, 2009; Young et al., 1997). Gittell and colleagues argue that effective coordination is obtained via good quality communication routines which enhance common knowledge, share goals, social ties and trust among staff (Gittell et al., 2008). Communication among staff must be frequent, timely, accurate and problem‐solving. Gittell and colleagues argue that such a *relational* approach to coordination through communication and collaboration routines is particularly relevant in work settings such as nursing homes that are characterized by high levels of task interdependence, uncertainty and time constraint. In the following, we will briefly explain what is meant by task interdependence, uncertainty and time constraints in the context of nursing homes, before we outline our research questions.

According to Gittell, (2006), Gittell et al. (2008), a low task interdependence means that employees do not have to confer with each other before carrying out a task autonomously, whereas a high task interdependence in a given work environment means that employees have to communicate with each other and collaborate to be able to get the tasks done. Thus, as Gittell et al. (2008, p. 156) point out, in long‐term care, “interdependencies are not the simple sequential handoffs found on production lines, but rather are iterative, requiring feedback among staff as new information emerges regarding a given resident.” *Uncertainty* further affects the need for relational coordination: When there is little uncertainty, the work process can be planned and there is little or no need to coordinate efforts to changing conditions. On the other hand, when uncertainty is high, employees must take heed of changes affecting their own work as well as others’ work (Gittell et al., 2008, pp. 155–156). Regarding the last point, Gittell et al. (2008, p. 156) argue that *time constraints “*exacerbate the effects of both interdependence and uncertainty, leaving little slack in the system and placing a premium on responsiveness.”

In this article, we will explore how the relational coordination approach may be useful to understand how communication and collaboration challenges are talked about and dealt with in two strategically selected nursing homes in Norway. Thus, in this article we explore the following questions: What communication and collaboration challenges are experienced among staff members? How are these challenges negotiated and dealt with? How can relational coordination be used as an analytical lens to shed light on the differences and similarities identified in the two nursing homes?

The article proceeds as follows: We start by describing the research method and procedures for the study before we present and discuss our findings in light of Gittels’ perspective on relational coordination. Finally, we conclude that **e**fforts by management to build competence, shared knowledge and frequent communication among staff may increase successful coordination and improve staff relations at the workplace.

## 3 METHOD

### 3.1 Design

This research project is based on a qualitative study For this article, we have compared and analysed data stemming from 31 (17 + 14) qualitative interviews with staff and management in two nursing homes in Norway.

### 3.2 Procedures

The first author was assisted by the Centre for Development of Institutional and Home Care Services (USHT) in contacting nursing homes for this study. Two nursing homes known to have a large share of multi‐ethnic staff accepted the invitation to take part in the study. The first author arranged a meeting with the managers and their staff to inform them about the project, answer questions and discuss how and when qualitative interviews could be organized. A list of potential participants for qualitative interviews was prepared together with the managers of both nursing homes. The main criterion when inviting potential interviewees was a wish for variation: hence we recruited interviewees with minority and majority backgrounds, with long and short experience from LTC, as well as staff members from all segments ranging from RNs with supplementary specializations employed full time, to auxiliary nurses and assistants in temporary and part time positions. All employees who were invited for an interview agreed and many clearly appreciated the opportunity to talk about communication and collaboration challenges in multicultural staff groups. An interview guide was drafted based on insights from previous studies on communication and cooperation issues in nursing homes as well as on immigrant care workers.

The interviews were carried out in the nursing homes, either in an office made available for the research project or in any other available room where the interview could be carried out undisturbed. Some interviews were carried out prior to or after a work shift, while others were conducted during the shift if other staff were available to take over the tasks and responsibilities of the interviewee. The recordings from the interviews lasted from 38 to 75 min, depending on how much information the participants wanted to share.

### 3.3 Participants

For this study, 31 members of staff were interviewed. Altogether, 17 of these were from Nursing home A and 14 from Nursing home B.

#### 3.3.1 Nursing home A

Nursing home A, located in a municipality of ~30.000 inhabitants in Eastern Norway, had 26 residents distributed among smaller wards. In total, the nursing home had ~50 employees in various positions and 17 were invited and agreed to be interviewed. The nursing home had a very low turnover, as both the manager as well as core staff in key positions such as the RNs and auxiliary nurses had been employed in the nursing home for nearly 15 years. In the municipality, the nursing home had a good reputation for high‐quality services. At the time of the survey, the 24 residents comprised women and men ranging in age from the mid‐sixties to the late nineties. Nearly, all the residents had a diagnosis of dementia (either as the main diagnosis or as an additional diagnosis), while a few had a Parkinson's disease diagnosis. The nursing home had a “homelike feeling” with attractive, light and nicely decorated common rooms and a lovely garden accessible for the residents.

#### 3.3.2 Nursing home B

Nursing home B, located in a municipality of ~15.000 inhabitants in Northern Norway, had 32 residents distributed among smaller wards. The total number of staff is ~90 and 14 were invited and agreed to be interviewed. The nursing home had a manager that had been recently recruited. Although some key staff had been employed for many years at the time of the study, several expressed interest in trying to find employment elsewhere. Approximately half of the residents were somatic frail elderly, while the other residents lived with advanced dementia. Challenging behaviour was common on a daily basis. The management was trying to group residents with a similar level of functioning together, but this was difficult as the health conditions of residents varied over time.

### 3.4 Data collection and analysis

All interviews were audiotaped and transcribed verbatim. The transcripts consisted of ~180 pages. In line with thematic analysis, the transcripts were read several times to gain a thorough overview of the data (Braun & Clarke, 2006). Sections relevant to the topic of communication and collaboration challenges and how these were dealt with (coordination strategies) were identified. The data were coded in the following themes: “types of communication challenges,” “principles of care work,” “prioritization of tasks” and subthemes addressing how challenges were negotiated. The subthemes that emerged were “meetings and shared knowledge,” “common aims and respect” and “resolving disagreements.” In the analysis, we compared staff interviews across position as well as within and across institutions.

## 4 ETHICS

All participants were informed about the study at a staff meeting. The staff members were encouraged to ask questions and express their opinion on the theme of the study and to discuss their willingness to participate. Managers and staff members found the study highly relevant and agreed to participate. The study was accepted by the Regional Ethic Committee and approved by the Norwegian Social Science Data Services (reference number 53138). For the interviews, all participants provided their informed consent to participate and were informed about their right to withdraw from the study without stating a reason.

## 5 RESULTS

### 5.1 Communication challenges among staff

Most of the staff members from both nursing homes said they sometimes experienced difficulties in their communication with other colleagues. Ethnic Norwegian care workers said that some staff with a minority background sometimes pretended to understand a message only to discover later that this was not the case. An ethnic Norwegian nurse who had worked at the nursing home for many years stated:

“Not everyone speaks good Norwegian when they start here. It can be a bit difficult for us … Something as simple as saying what needs to be done and giving instructions …And you ask if they've understood. ‘Yes, yes’, they say … But then it often turns out that they haven't understood anything at all. I wonder if they're reluctant to ask again?”

Another challenge in staff communication relates to understanding the more specialized terminology in nursing homes. This could be communication about illnesses, conditions and medication, observation of the patients and how to verbalize changes in their condition, etc.

### 5.2 Understanding the principles of care work

The managers in the two nursing homes claimed that in general their staff understood the principles of the care work, especially those who had been working for many years and had acquired considerable work experience. However, an issue of concern among the management in both nursing home A and B was if and how workers with minority background were able to use their own discretion in situations that required a change of approach to, for example, feeding, or changing the diaper when this deviated from the original plan. They explained that the needs and health status of some residents could change from one hour to the next which entailed deviating from instructions given earlier on the same day. The manager in nursing home B described how “unskilled” assistants with a minority background were especially reluctant to deviate from care plans and schedules:

“It's got to do with understanding what comes first. But many of those with a minority background who come here to work are very conscientious so if the schedule says ‘Shower’, that maybe means that they will carry out this task without thinking that it might be smart to do it another day. In other words, they do their job, that's for sure [the informant chuckles].”

Another issue of concern was how the term “reablement” (which represents a service approach where focus has shifted from passive reception of help to activity and self‐sufficiency) should be understood. In both nursing homes, the management and core staff wanted the residents to do as much as they could themselves. If a resident was able wash her face, she should be encouraged to do so, even if that could take a little longer time than if a care worker just washed her face for her. Similarly, if a resident was able to spread the butter and put jam on a slice of bread for breakfast, he should be supported to do so rather than having one of the care workers doing it for him. Whether or not this “everyday rehabilitation” approach was a good thing was a debated issue among staff in both nursing homes. An experienced minority worker noted how upset she had been at first when she noticed that the residents, as she saw it back then, did not get enough help from the care workers in the nursing home:

“…I thought: Why don't they [staff at the nursing home] help them? They just put the food in front of them and he [a resident]… makes a mess and spills things and so on. But then I thought… when I took that course [on everyday rehabilitation], that they must feel they can manage things themselves. And that's very, very important for the patient. And I didn't know about that before.”

Another participant also claimed it was frustrating to be told what not to do when she witnessed that the residents needed a lot more help than she as an employee was expected to give. For this reason, she explained, she preferred to work alone, so she could feel that she did a “proper” job:

“I like best to work on my own … When I work with others, I’m always being instructed to do this and that and no more! But I like to do a proper job! I like to tidy and clean and fix things … but when I’m with others [staff] they say: ‘Wash the floor!’ But when I’m alone, I do a lot more.”

### 5.3 Carrying out and prioritizing care tasks

Both nursing homes included in this study had a similar approach to the division of tasks among staff. The formal educational background of staff members was normally given relatively little attention, except for the few tasks that were regulated as the sole responsibility of RNs. This included cleaning of wounds, taking blood samples and distributing medicines to the units. Apart from these tasks, all staff were expected to carry out most of the tasks, such as helping residents in the morning and evening with daily care tasks, helping around the kitchen and at mealtimes as well as organizing activities and socializing with the residents.

At both nursing homes, discussions had occurred between staff about how to prioritize among the different care tasks. In the interviews, there was a general opinion among the ethnic Norwegian staff that the immigrant workers preferred the general responsibility for the cleanliness and appearance of the unit. This implied being responsible for what was termed as “red tasks” in one nursing home. These tasks included preparing and serving food, setting the tables, cleaning the dishes after meals and washing tablecloths and linen if needed.

The leader in one nursing home explained that these tasks might be clearer and more direct and it gives a feeling of “working.” She claimed that the other tasks in the ward (referred to as “green tasks” or “working from the sofa‐group”) such as taking care of the residents’ social needs, maintaining a good atmosphere in the unit, being attentive to the needs of the residents and resolving any conflicts before they escalated were more demanding tasks because the procedures for these tasks were less clear:

“It's difficult to be the person who holds the group of residents together, being in the “sofa‐group” as we call it… And the weakness here is the language, the culture and their inner feeling that they [staff with immigrant background] must work. And sitting down is not work to them …that's the attitude.”

As we have seen above, communicating with residents was described as demanding by all interviewees and at times very challenging. However, some immigrant workers regarded the work of the “sofa group” as somehow less valuable or important as doing cleaning and food preparation. A staff member from East‐Europe claimed initially in the interview that she liked to do all tasks and that she had no preferences. However, when asked directly if she liked to sit down with the residents in the sofa group, she said:

“No, absolutely not! I don't sit there for long. I like to work as I normally do! I like to do things. I’m not someone who can sit in a sofa for a long time. What's special about people with dementia is that if they see that we just stand up and leave, they get confused, so you have to sit down again to get them to calm down and chat a bit longer.”

However, the nurse claimed that discussions and disagreements in teams could arise when staff members valued the importance of the care tasks differently. This turned out to be a source of disagreement related to works tasks between staff sharing shifts:

“One person may feel that the other has wrong priorities. While some people think it's important that the sterilization room is clean, others prioritize spending time chatting in the sofa, finding activities for the residents …Yes, focusing on a good social environment.”

The staff generally agreed that it was challenging to keep an overview of a group of residents with varying wishes in addition to acute issues that could arise. An ethnic Norwegian auxiliary claimed that priorities also had to do with feeling successful at work:

“They [immigrant workers] clearly think that they do a better job when they're actively cleaning or doing direct care work, right? At least I’m working, they think and they're proud of working a lot.”

This often meant that ethnic Norwegian workers looked after the social needs of the residents and contributed to a good atmosphere in the ward while the immigrant workers were cleaning and organizing meals.

### 5.4 MANAGING CHALLENGES: Frequent meetings provide shared knowledge

Although the challenges were experienced and described in similar ways in the two nursing homes taking part in this study, they were handled by the management in each of the nursing homes in very different ways.

The relationships between staff members in the two nursing homes were different. The staff at nursing home A had considerably more contact and communication and the manager emphasized the importance of regular meetings. They had a large, dual purpose room that was used both for meetings and having lunch. The room was located centrally in the nursing home, overlooking the entrance as well as providing a good overview of the residents’ wards located on either side. The manager's office was located next to the meeting room. She always ate lunch together with the members of staff and led all the morning meetings herself. The leader explained in the interview that she had put a firm emphasis on frequent communication, always making sure that as many staff members as possible were present at meetings:

“We have meetings daily … we have a morning meeting every day from 08.00– 08:30. Previously we tried to shorten this to just a very brief meeting. Everyone was then supposed to go online and read up on what was there … then everyone should return to their own work. After just a few days, no one said anything! We ended up with poor communication because we were a bit silenced by this ….”

The manager pointed out that even though the information about the daily tasks and the residents were available to her employees electronically, they missed the fellowship and interaction of the meetings:

“So, we reversed this and started up the meetings again … And this fellowship, the fact that we sit and reflect on things together and bring up things we wonder about, it's so important. And in addition, we give information when people are gathered.”

This stands in sharp contrast to nursing home B. The manager organized her staff in two teams working in two separate wards. The residents were grouped into a mild and a severe dementia unit. The residents at the severe dementia unit needed constant monitoring. Episodes of residents attacking each other or attempting to destroy furniture and equipment happened regularly. Staff members were exposed to spitting, pinching, scratching and verbal assaults. The need for constant monitoring made it more difficult to gather staff at meetings. The meeting room in one of the wards had no view into the unit where the residents’ common room was located. The manager claimed that she had one weekly meeting with the staff, but even at this meeting it was hard to gather all members of staff. Sometimes, if special situations occurred with residents, the staff were explicitly asked to attend the meeting even if they were not on duty at the allocated time:

“I have meetings with staff in both wards on a weekly basis. My staff on afternoon shifts get paid time off if they attend. Then we talk about cooperation, residents and care plans. In addition, I have encouraged my staff to evaluate each shift, but I honestly don't know how often this happens.”

The manager at this unit encouraged her staff to evaluate every single shift but left it to the staff themselves to decide if they should meet or not. If there had been episodes or disagreements on shifts that staff wanted to evaluate, these evaluations were cancelled unless all staff members on the shift were able to be present. In interviews, staff members claimed it was unfair to evaluate a shift if not everybody was present to voice their opinion. The staff claimed that evaluations were most necessary if something unexpected had happened during the shift in relation to residents or failure among staff to cooperate. At the time of the interviews, several episodes occurred where members of staff asked to evaluate a shift, but this was not done because it was difficult to gather everyone on the same shift.

In addition to regular meetings ensuring that all staff got comprehensive information, the manager at nursing home A was dedicated to explaining care tasks and was aware that everything might not be fully understood by immigrant workers. Some tasks were at risk of just being “counted in” without being visualized or described anywhere. In discussing the content of the “green tasks” in nursing home A as presented above, a nurse stated:

“I don't think there's any written description of what work to improve the social environment consists of or initiating social interaction or organizing a get‐together … no, I don't think there's any written description anywhere. But I would say that it's sort of understood. Just sitting there talking to residents, no, there's nothing about that anywhere…”

This awareness of what is included or implied in various care tasks helped staff to reduce misunderstandings.

### 5.5 Shared goals and mutual respect

In nursing home A, the manager acknowledged the communication difficulties that her employees were experiencing and she repeatedly held courses in how to communicate with residents with dementia in particular. Staff in both nursing homes found it stressful to be around residents who were screaming, making difficult demands or even using manipulation to get their own way. As a result of courses and frequent communication, the staff in nursing home A had found a common approach for how to handle difficulties and had established an arena where they could discuss experiences. It helped to prevent disagreements. For example, all staff members agreed to consciously use few words and give clear short messages to residents with dementia. They had also built a deeper understanding for each other whenever a staff member experienced communication challenges with a resident. If one staff member was having difficulty carrying out care work with a resident, there was a low threshold for asking a colleague to step in.

In nursing home A, challenges and difficulties in the ward with residents or between staff were also discussed with the manager or in meetings. The staff shared thoughts and the manager was especially concerned that also the immigrant workers should voice their thoughts and concerns. The manager encouraged staff with immigrant background to ask questions and to request that ethnic colleagues repeat the instructions or procedures if tasks were not properly understood. The nursing home developed a tolerance for questioning, and it made it easier for staff to ask for clarifications. A Norwegian nurse said:

“Sometimes I might ask them [minority background staff] to repeat what I said and then it's easy to know whether they've understood or not. So, then we do it again until they have understood.”

### 5.6 Resolving disagreements

An immigrant care worker explained that she benefitted from the atmosphere in nursing home A where she could ask for help and clarification. She argued that the most important thing she had learnt in the nursing home was the Norwegian language and the goodwill and patience of her colleagues:

“Practise with colleagues and familiarize yourself … because they [the colleagues] show me things and talk to me and then I started to learn it [the work tasks for the job].”

In nursing home B, however, communication and language learning were discussed less openly and the manager strove to make staff members attend meetings. Staff in nursing home B experienced more situations involving miscommunication and distrust between staff members. It was especially difficult when staff teams of two were to cooperate on a shift with residents who refused to receive care. There had been several episodes of ethnic Norwegian staff refusing to carry out care shifts with unskilled immigrant workers. In one situation, an ethnic Norwegian care worker did not feel that she could trust the co‐worker on the shift**—**an immigrant care worker who had a part‐time position while taking formal education as a care worker.

The combination of a failure to attend meetings and less room for resolving disagreements and building a good working atmosphere resulted in lack of shared knowledge and goals among staff vis‐à‐vis the care work. Ultimately, this led to considerably less mutual respect among staff members, where staff excluded each other instead of sharing knowledge and learning from each other.

## 6 DISCUSSION

Good communication and common understanding of the care work was a prerequisite for successful coordination and cooperation. Staff in both nursing homes described a panorama of communication challenges related to residents with complex physical and psychological health problems. Communication with residents was, however, more than a purely linguistic issue. It also required cultural understanding of the care practices and knowledge of how to respond to a variety of health problems, for example dementia. It has long been known that thoughts about illness and health as well as care and treatment vary from society to society (Helman, 2007; Kleinman, 1980). Healthcare performance is attached to variations in culture and social norms (Zhou et al., 2011). In a multicultural working community, established ideas about how things should be done “right” and what the care profession “is” are challenged (Rogstad and Solbrække, 2012, p. 319) and need to be learnt. For example, our findings that staff members have divergent understandings of the principles of care work and how to prioritize care tasks reflect cultural variation and diversity. The management in nursing home A acknowledged this and worked to build common understanding in frequent meetings, sharing knowledge and fostering mutual respect among all members of staff. This approach led to a lower threshold for asking questions and discussing care practices. It benefitted all staff members, but especially immigrant workers who could otherwise feel hesitant about asking questions. Nursing home B was less successful in bringing staff together. Few meetings and lack of room for communication resulted in less sharing of information and knowledge, as well as less trust among staff.

In nursing homes, coordination becomes especially important as *task interdependence* exists between different members of staff who collaborate in the provision of care for residents. While individual members of staff carry out many care tasks autonomously, other tasks require simultaneous action from two or several staff members. An example of a task carried out autonomously by one staff member is drug administration, while examples of tasks that require simultaneous action include providing personal care to residents who resist services or preparing for a meal and gathering the residents together for food and medicines. As Gittell et al., 2008, p. 156) point out, in long‐term care, “interdependencies are not the simple sequential handoffs found on production lines, but rather are iterative, requiring feedback among staff as new information emerges regarding a given resident.” This underlines the constant need for communication and understanding among staff with various backgrounds and the importance of strengthening multicultural competence and diversity management.

Our results also bring attention to how the increased complexity and severity of residents’ health conditions further challenge cooperation and communication. Research has drawn attention to the potential adverse impacts of quality in care due to communication challenges (Egede‐Nissen et al., 2013; Habermann & Stagge, 2010). The variability of the physical and mental health of elderly residents in contemporary nursing homes activates *uncertainty*. Residents’ health conditions can change quickly and within hours. This means that all staff must be mindful of residents’ changing conditions and communicate changes to each other so that responses are appropriate and coordinated. Simultaneously, time constraints exist because of restrictions on the availability of carers. All staff need to respond to care recipients’ needs in a timely fashion to avoid negative clinical outcomes (Gittell et al., 2008).

Our study further shows that both management styles and physical infrastructure are decisive in building successful relations at the nursing homes. In nursing home A, good routines for frequent communication were pivotal for sharing information about residents and the meetings took place in a large room centrally located in the nursing home. It made it easier for all staff to attend meetings because they could keep an eye on the residents simultaneously.

Moreover, shared knowledge and building a culture for fostering common understanding among staff can improve quality of care (Gittell, 2009; McDaniel & Driebe, 2001). Staff in nursing home A demonstrated care tasks to the immigrant workers regularly and had routines for ensuring that care tasks were understood. Our findings support research stressing that immigrant healthcare workers are in need of support from both management and staff for successful transition (Choi et al., 2019). The process of accommodating differences in care practices is found to be lengthier than some transition programmes allow for (Brunton & Cook, 2018). Supportive leadership encouraging good communication and tolerance for all workers builds multicultural workplaces fostering ethnic equality (Janssens & Zanoni, 2014). More successful teams are likely to be built when learning the language in the host country is seen as an ongoing process met with tolerance among all members of staff in comparison to workplaces where it is expected that minority workers are fully in command of the new language when they start (Munkejord & Tingvold, 2019).

Nursing home A in our study accepted the need for continued language training to cope with communication challenges in relation to residents and staff members. It also helped the migrant workers to understand the underlying principles of care practices. This approach is consistent with studies demonstrating the need for a supportive learning environment in an increasingly complex sector (Anvik et al., 2020) and for strengthening clinical supervision for foreign educated nurses to increase patient safety competencies (Viken et al., 2018). To create good intergroup communication, equal status in the situation, common goals, inter group cooperation and support from the authorities are essential (Allport, 1954; Munkejord, 2019; Pettigrew & Tropp, 2013).

## 7 CONCLUSION

Nursing homes serve residents with complex and rapidly changing care needs. The workplace is characterized by high pressure with many staff members with different backgrounds and qualifications working together. These factors challenge cooperation and coordination among staff. In this complex terrain, our study shows that management that rewards frequent communication and meetings among all staff members may be more successful in building trust and shared goals among staff. Acknowledgment of the need for continued learning for immigrant workers and ensuring support from other members of staff are of key importance in building good staff teams in the future.
